# Targeting the Eph/Ephrin System as Anti-Inflammatory Strategy in IBD

**DOI:** 10.3389/fphar.2019.00691

**Published:** 2019-06-17

**Authors:** Andrea Grandi, Irene Zini, Simone Palese, Carmine Giorgio, Massimiliano Tognolini, Francesco Marchesani, Stefano Bruno, Lisa Flammini, Anna Maria Cantoni, Riccardo Castelli, Alessio Lodola, Antonella Fusari, Elisabetta Barocelli, Simona Bertoni

**Affiliations:** ^1^Food and Drug Department, University of Parma, Parma, Italy; ^2^Department of Veterinary Sciences, University of Parma, Parma, Italy

**Keywords:** TNBS-induced colitis, DSS experimental colitis, splenocytes culture, ephrin-B2, EphB4, EphB1-Fc

## Abstract

Besides their long-known critical role in embryonic growth and in cancer development and progression, erythropoietin-producing hepatocellular carcinoma type B (EphB) receptor tyrosine kinases and their ephrin-B ligands are involved in the modulation of immune responses and in remodeling and maintaining the integrity of the intestinal epithelial layer. These processes are critically involved in the pathogenesis of inflammatory-based disorders of the gut, like inflammatory bowel diseases (IBDs). Accordingly, our aim was to investigate the role of the EphB/ephrin-B system in intestinal inflammation by assessing the local and systemic effects produced by its pharmacological manipulation in 2,4,6-trinitrobenzenesulfonic acid (TNBS)- (Th1-dependent model) and dextran sulphate sodium (DSS)- (innate response model) induced colitis in mice. To this purpose, we administered chimeric Fc-conjugated proteins, allegedly able to uni-directionally activate either forward (ephrin-B1-Fc) or reverse (EphB1-Fc) signaling, and the soluble monomeric EphB4 extracellular domain protein, that, simultaneously interfering with both signaling pathways, acts as EphB/ephrin-B antagonist.The blockade of the EphB/ephrin-B forward signaling by EphB4 and EphB1-Fc was ineffective against DSS-induced colitis while it evoked remarkable beneficial effects against TNBS colitis: it counteracted all the evaluated inflammatory responses and the changes elicited on splenic T lymphocytes subpopulations, without preventing the appearance of a splice variant of ephrin-B2 gene elicited by the haptenating agent in the colon. Interestingly, EphB4, preferentially displacing EphB4/ephrin-B2 interaction over EphB1/ephrin-B1 binding, was able to promote Tumor Necrosis Factor alpha (TNFα) release by splenic mononuclear cells *in vitro*. On the whole, the collected results point to a potential role of the EphB/ephrin-B system as a pharmacological target in intestinal inflammatory disorders and suggest that the therapeutic efficacy of its blockade seemingly works through the modulation of immune responses, independent of the changes at the transcriptional and translational level of EphB4 and ephrin-B2 genes.

## Introduction

Eph receptors (erythropoietin-producing hepatocellular carcinoma) belong to the largest family of tyrosine kinases receptors (RTKs), divided in A (EphA1-8, 10) and B classes (EphB1-4, 6), according to structural features and binding affinities for their ligands, cell surface-bound ephrins (Eph receptors interacting proteins) (Pasquale, 2005). Eph receptors interact with ephrins on neighboring cells, generating cell contact-dependent bidirectional signaling that affects both the Eph- and the ephrin-bearing cells (Lodola et al., 2017). Specifically, forward signaling is activated in Eph-bearing cells by ephrin binding, whereas reverse signaling is activated in ephrin-bearing cells by Eph binding (Barquilla and Pasquale, 2015).

The Eph/ephrin signaling regulates a wide range of cellular functions, encompassing cell proliferation and survival, cell shape, and migration (Kania and Klein, 2016). Besides its long known critical role in embryonic growth and in cancer development and progression, recent findings have highlighted its involvement in cell adhesion-based responses, in the modulation of immune responses, and in remodeling and maintaining the integrity of the epithelial layer (Funk and Orr, 2013; Perez White and Getsios, 2014). In particular, the interaction between EphB4 on leukocytes and endothelial ephrin-B2 regulates endothelial activation, leukocytes adhesion, and transmigration (Pfaff et al., 2008); moreover, ephrin-B1, ephrin-B2, and EphB4 are involved in T-cell development and activation (Kawano et al., 2012; Jin et al., 2014), in the organization of stem cell compartments and in the ordered migration of epithelial cells along the intestinal villus axis (Perez White and Getsios, 2014). Collectively, the EphB/ephrin-B system appears to affect several cellular targets critically involved in the pathogenesis of inflammatory-based disorders, like inflammatory bowel diseases (IBDs).

IBDs are chronic inflammatory disorders of the gastrointestinal (GI) tract, characterized by an aberrant immune response against luminal antigens. Crohn’s disease (CD) and ulcerative colitis (UC) are the two principal types of IBD: they show a relapsing–remitting course and are characterized by impaired intestinal epithelial barrier and abnormal T-cell responses (Kaser et al., 2010). Interestingly, the expression of B-type Eph/ephrins in IBD patients is altered: in particular, ephrin-B2 messenger RNA (mRNA) is over-expressed in the gut mucosal lesions of CD patients (Hafner et al., 2005).

Despite this intriguing preliminary evidence, the intervention of EphB/ephrin-B in colitis has remained basically unexplored till now. Hence, our primary aim was to investigate the role of EphB/ephrin-B system in intestinal inflammation by assessing the local and systemic effects produced by pharmacological manipulation of EphB/ephrin-B pathway in two distinct chemically induced murine models of IBD: acute 2,4,6-trinitrobenzenesulfonic acid (TNBS)-induced colitis, a Th1-mediated model of intestinal inflammation, and dextran sulphate sodium (DSS)-induced colitis, characterized primarily by activation of innate immune responses (Kiesler et al., 2015). To this purpose, we administered chimeric Fc-conjugated proteins—allegedly able to uni-directionally activate forward signaling and to block the reverse one (ephrin-B1-Fc), or vice versa, to activate reverse signaling and to block the forward one (EphB1-Fc) (Barquilla and Pasquale, 2015)—and soluble EphB4 extracellular domain, which, being a monomer devoid of Fc fragment, is reported to simultaneously interfere with both forward and reverse signaling pathways and to behave as EphB/ephrin-B antagonist (Kertesz et al., 2006). The supposed functional effects of the administered recombinant proteins are reported in **Table 1** for the sake of clarity. As indicators of colitis severity, we evaluated both clinical and inflammatory markers in mice exposed to TNBS or DSS. Moreover, to assess whether the intestinal inflammatory process induced by TNBS and the beneficial action, provided by monomeric EphB4, were associated with changes in the expression levels of ephrin-B2 and of its preferential ligand EphB4 in the colon, their mRNA and protein levels were determined in normal mice and in vehicle- and drugs-treated colitic mice. Finally, to clarify the mechanism of action of the EphB/ephrin-B antagonist, we tested the effects of monomeric EphB4 on the activation of splenic mononuclear cells through *in vitro* assays.

**Table 1:** Purported functional effects of administered recombinant proteins ephrin-B1-Fc, EphB1-Fc, and EphB4.

Recombinant protein	Target	Effects	
		Forward signaling	Reverse signaling
ephrin-B1-Fc	EphB	↑	↓
EphB1-Fc	ephrin-B	↓	↑
EphB4	ephrin-B	↓	↓

↑, Increased; ↓, Decreased.

Adapted from Barquilla and Pasquale, 2015.

## Materials and Methods

### Animals

Female C57BL/6 mice (8–12 weeks old) (Charles River Laboratories, Calco, Italy), weighing 20 to 24 g, were housed five per cage in identical conditions at least 7 days before experiment started. They were maintained under standard conditions at our animal facility (12:12 h light–dark cycle, 22°C to 24°C, food and water available *ad libitum*). All the experimental procedures and suppression by CO_2_ inhalation were performed between 9:00 am and 12:00 am. All appropriate measures were taken to minimize pain or discomfort of animals. Animal experiments were performed according to the guidelines for the use and care of laboratory animals, and they were authorized by the local Animal Care Committee “Organismo Preposto al Benessere degli Animali” and by Italian Ministry of Health “Ministero della Salute” (DL 26/2014). Animal studies were reported in compliance with the Animal Research: Reporting of In Vivo Experiments (ARRIVE) guidelines (Bramhall et al., 2015).

### Induction of Colitis

Seven days before intrarectal (i.r.) instillation, animals were subjected to skin sensitization through cutaneous application of 50 μl of a 10% (w/v) TNBS solution in 50% ethanol. After 20 h of fasting with free access to water containing 5% glucose, a 10-cm-long PE-50 tubing attached to a tuberculin syringe was inserted 4 cm into the anus of mice. The i.r. administration of the same volume and concentration of TNBS solution applied during skin sensitization was performed in anaesthetised mice (isoflurane 2%) kept in a vertical head-down position for 3 min to avoid leakage of the intracolonic instillate. Animals were suppressed by CO_2_ inhalation three days after TNBS instillation. The TNBS-induced colitis model with previous skin sensitization was chosen to reproduce a model of colitis characterized by the prominent activation of the adaptive immune system (te Velde et al., 2006).

DSS colitis was induced by free access to 3% w/v DSS (MW, 36–50 kDa) solution in drinking water for 5 days. Animals were suppressed by CO_2_ inhalation on day 8 (Waldner and Neurath, 2009).

### Experimental Design

Pharmacological treatments started 8 h after the exposure to the colitogenic agent (TNBS i.r.; DSS in drinking water) and were applied for 3 days in TNBS colitis and for 5 days in DSS colitis.

Mice were assigned through block randomization to the normal group (N), comprising mice intra-rectally inoculated with 50 μl 0.9% NaCl (saline solution) (for TNBS colitis, *n* = 30) or receiving drinking water (for DSS colitis, *n* = 15) and administered 10 mL/kg/day saline subcutaneously (s.c.), or to the following experimental groups of colitic mice: saline [CNT, 10 mL/kg s.c., *n* = 20 (DSS); *n* = 40 (TNBS)], EphB1-Fc [30 µg/kg s.c., *n* = 6 (DSS); *n* = 10 (TNBS)], ephrin-B1-Fc [17 µg/kg s.c., *n* = 5 (DSS and TNBS)], EphB4 [20 µg/kg s.c., *n* = 6 (DSS); *n* = 15 (TNBS)], IgG1-Fc (10 µg/kg s.c., *n* = 6 (negative control for TNBS)], cyclosporine A (CsA) [25 mg/kg/die intraperitoneal (i.p.), *n* = 7 (positive control for DSS)]. **Table 2** briefly resumes the characteristics of the experimental groups. The dosage and route of administration of EphB4 and CsA were chosen on the basis of preliminary experiments and previous investigations (Satoh et al., 2009), and doses of ephrin-B1-Fc, EphB1-Fc, and IgG1-Fc equimolar to EphB4 were applied. The study was performed using experimental blocks composed by 10 or 12 mice that were randomly assigned to four or five groups of treatment (*N* and CNT were present in each experimental block), each one encompassing two animals.

**Table 2:** Experimental design with characteristics of each experimental group.

Groups	TNBS colitis			DSS colitis		
	Colitogenic agent	Treatment	N	Colitogenic agent	Treatment	N
N	Saline	Saline s.c.	30	Water	Saline s.c.	15
CNT	TNBS	Saline s.c.	40	DSS	Saline s.c.	20
EphB1-Fc	TNBS	EphB1-Fc s.c.	10	DSS	EphB1-Fc s.c.	6
Ephrin-B1-Fc	TNBS	Ephrin-B1-Fc s.c.	5	DSS	Ephrin-B1-Fc s.c.	5
EphB4	TNBS	EphB4 s.c.	15	DSS	EphB4 s.c.	6
IgG1-Fc	TNBS	IgG1-Fc s.c.	6	DSS	n.d.	n.d.
CsA	TNBS	n.d.	n.d.	DSS	Cyclosporine A i.p.	7

N, number of mice in each experimental group; n.d., not determined; TNBS, 2,4,6-trinitrobenzenesulfonic acid; DSS, dextran sulphate sodium; s.c., subcutaneously administered; i.p., intraperitoneally administered.

Due to possible seasonal variability, *N* and CNT mice were repeated periodically all over the study to verify the attainment of a constant degree of colitis severity with respect to physiological conditions, thus explaining the bigger size of *N* and CNT experimental groups with respect to the other groups. Accordingly, each group of animals was randomly subdivided in two subgroups: colons excised from each subset was reserved either for histological analysis or for myeloperoxidase (MPO) activity determination, reverse transcription polymerase chain reaction (RT-PCR)/Western blotting assays, and for cytokine assays.

### Evaluation of Inflammatory Responses

Disease Activity Index (DAI) was measured daily throughout the experimentation. Immediately after suppression, the macroscopic damage of colonic mucosa was assessed as macroscopic score (MS). The wet weight and the length of each colon were measured, and the weight/length ratio was considered as disease-related intestinal wall thickening. Colon, lungs, spleen, and mesenteric lymph nodes were collected for subsequent microscopic, biochemical, or flow cytometry analyses.

#### Disease Activity Index (DAI)

The severity of experimental colitis was estimated as DAI, a score assigned daily on the basis of body weight loss, stool consistency, and rectal bleeding, by unaware investigators in accordance to Cooper’s modified method (Cooper et al., 1993). The scores were quantified as follows: stool consistency: 0 (normal), 1 (soft), 2 (liquid); body weight loss: 0 (<5%), 1 (5–10%), 2 (10–15%), 3 (15–20%), 4 (20–25%), 5 (>25%); rectal bleeding: 0 (absent), 1 (light bleeding), 2 (heavy bleeding).

#### Macroscopic Damage of Colonic Mucosa (MS)

After suppression, the colon was explanted, opened longitudinally, gently flushed with saline solution, and MS was assessed through examination of the mucosa by an investigator unaware of the treatments applied. MS was determined according to previously published criteria (Rapalli et al., 2016) as the sum of scores (max = 12) attributed as follows: presence of strictures and hypertrophic zones (0, absent; 1, one stricture; 2, two strictures; 3, more than two strictures), mucus (0, absent; 1, present), adhesion areas between the colon and other intra-abdominal organs (0, absent; 1, 1 adhesion area; 2, 2 adhesion areas; 3, more than 2 adhesion areas), intraluminal hemorrhage (0, absent; 1, present), erythema (0, absent; 1, presence of a crimsoned area < 1 cm^2^; 2, presence of a crimsoned area > 1 cm^2^), and ulcerations and necrotic areas (0, absent; 1, presence of a necrotic area < 1 cm^2^; 2, presence of a necrotic area > 1 cm^2^).

#### Colonic Length and Thickness

The length of colon and its weight were measured to assess deposition of fibrotic material and muscular contraction elicited by colitis induction; weight/length ratio was calculated to assess colon thickness, according to previously published criteria (Grandi et al., 2017).

#### Colonic and Pulmonary Myeloperoxidase (MPO) Activity Assay

MPO activity, a marker of tissue granulocytes accumulation, was determined as described elsewhere (Vivo et al., 2017). Briefly, colonic and lung samples were weighed and homogenized in ice-cold potassium phosphate buffer (100 mM, pH 7.4) containing 1 µg/mL aprotinin and centrifuged for 20 min at 12,500 RCF at 4°C. Pellets were re-homogenized in five volumes of ice-cold potassium phosphate buffer (50 mM, pH 6) containing 0.5% hexadecylthrimethyl-ammoniumbromide (HTAB) and 1 µg/mL aprotinin. Then, samples went through three cycles of freezing and thawing and were centrifuged for 30 min at 15,500 RCF at 4°C. 100 µL of the supernatant was allowed to react with 900 µL of a buffer solution containing 0.167 mg/mL o-dianisidine and 0.0005% H_2_O_2_. Each assay was performed in duplicate, and the rate of change in absorbance was measured spectrophotometrically at 470 nm (Jenway, mod. 6300, Dunmow, Essex, England). The sensitivity of the assay was 10 mU/mL, 1 unit of MPO being defined as the quantity of enzyme degrading 1 μmol of peroxide per minute at 25°C. Data were normalized with edema values [(wet weight − dry weight)/dry weight] (Moore-Olufemi et al., 2005) and expressed as U/g of dry weight tissue.

#### Colonic Interleukin-1β Levels

In N, CNT and EphB4-treated mice exposed to TNBS, colonic interleukin-1β (IL-1β) levels were determined using a commercially available Enzyme-Linked ImmunoSorbent Assay (ELISA) kit (IL-1β Mouse SimpleStep ELISA^™^ kit; Abcam Biochemicals^™^, Cambridge, UK). Samples were homogenized for 1 min in 700 µL of extraction buffer in accordance to the manufacturer’s protocols. Samples were then centrifuged for 30 min at 14,000 RCF, and the supernatant was collected. Total protein concentration was quantiﬁed using Pierce bicinchoninic acid (BCA) protein assay kit (ThermoFisher Scientiﬁc Inc., Waltham, MA, USA). Colonic concentrations of IL-1β were determined in duplicate in 100 µL samples: the absorbance was measured spectrophotometrically at 450 nm (TECAN Sunrise^™^ powered by Magellan^™^ data analysis software, Mannedorf, Switzerland). The assays sensitivity for IL-1β was 5 pg/mL. Results were expressed as pg/mg protein.

#### Colonic Histology

Colonic samples were harvested from normal mice (*n* = 3) and TNBS-treated animals administered with saline (*n* = 3) or EphB4 (*n* = 4), immersion-ﬁxed in 10% neutral buffered formalin overnight, dehydrated, and embedded in paraffin. For each sample, at least ﬁve transverse 5-µm sections were cut in the distal colon, stained with hematoxylin–eosin, and blindly examined in a light microscope (Nikon Eclipse E800). The histological damage was quantified using Bischoff’s modified method (Bischoff et al., 2009): the grade of mucosal destruction (0, normal; 1, mild; 2, moderate; 3, severe) and the degree of leukocytes inﬁltration in the lamina propria and submucosa (0, absent; 1, mild; 2, pronounced) were scored (maximum total score: 7). The average value of histological score was determined for each colon, pooled with those determined for colons of the other animals in the same experimental group, and the mean value ± SEM was calculated.

#### Evaluation of EphB4 and Ephrin-B2 Expression

##### Reverse Transcription Polymerase Chain Reaction (RT-PCR)

Total RNA was isolated from colonic samples of *N* and TNBS-induced colitic mice, either treated with saline or EphB4, through *Qiagen RNeasy Protect Mini Kit* (Qiagen, Hilden, DE) and quantified using Nanodrop ND-1000 (Thermo Fisher Scientific Inc, Waltham, MA). 1 µg of RNA was reverse transcribed into complementary DNA (cDNA) and amplified using OneStep RT-PCR kit (Qiagen, Hilden, DE), according to the manufacturer’s protocol. The following primer pairs, purchased by Life Technologies Italia (Monza, MB, Italy), were used:

- ephrin-B2, 5′-ACCCACAGATAGGAGACAAA-3′ (forward),
- 5′-GGTTGATCCAGCAGAACTTG-3′ (reverse);
- EphB4, 5′-AGCCCCAAATAGGAGACGAG-3′ (forward),
- 5′-GGATAGCCCATGACAGGATC-3′ (reverse);
- GAPDH, 5′-GACTCCACTCACGGCAAATT-3′ (forward),
- 5′-TCCTCAGTGTAGCCCAAGAT-3′ (reverse).

PCR was conducted for 36 cycles to amplify EphB4 and ephrin-B2 sequences according to Ogawa et al. (2006). The following conditions were used for amplification: EphB4: denaturation for 10 s at 94°C, annealing for 45 s at 53°C, and extension for 3 min at 68°C; ephrinB2: denaturation for 45 s at 94°C, annealing for 45 s at 53°C, and extension for 90 s at 72°C. PCR products were separated on 1% agarose gels, visualized with a ChemiDoc^™^ Imaging System (Bio-Rad, Berkeley, CA, USA) after staining with RedSafe^™^ (iNtRON Biotechnology, Seongnam, South Korea) and analyzed by means of Image Lab^™^ software, Bio-Rad Laboratories, Inc. version 6.0. Expression levels of EphB4 and ephrin-B2 mRNAs were determined from four to seven independent samples after normalization by reference to the signal intensity of the band of Glyceraldehyde-3-phosphate dehydrogenase (GAPDH) mRNA (PCR for 30 cycles, denaturation for 45 s at 94°C, annealing for 45 s at 93°C, and extension for 90 s at 72°C) obtained in the same specimen.

##### Western Blotting

Tissue lysates, obtained from colonic specimens excised from N, CNT, and EphB4 TNBS-treated mice, were subjected to sodium dodecyl sulfate polyacrylamide gel electrophoresis (SDS-PAGE). Gels were then electro-blotted onto a nitrocellulose membrane (Bio-Rad, Hercules, CA) using a semi-dry transfer apparatus (Thermo Fisher Scientific Inc, Waltham, MA) containing a mixture of tris-glycine transfer buffer and 20% methanol. The nitrocellulose membranes were then incubated in blocking buffer (bovine serum albumin 5%) for 1 h at room temperature and incubated overnight at 4°C with primary antibodies against EphB4 (catalog number: 20883-1-AP, lot number 00016411, Proteintech, Manchester, UK), ephrin-B2 (catalog number NBP1-84830, Novus Biologicals, Littleton, CO, USA) and β-actin (catalog number MA5-15739-BTIN, lot number RB233172, Thermo Fisher Scientific Inc, Waltham, MA). After washing three times with tris-buffered saline, the membranes were incubated with horseradish peroxidase-conjugated secondary antibodies (Thermo Fisher Scientific Inc, Waltham, MA) for 1 h at room temperature. Protein bands were visualized using a chemiluminescence detection kit (Thermo Fisher Scientific Inc, Waltham, MA) and analyzed through C-DiGit Blot Scanner (Li-Cor, Nebraska, USA). The signal intensity of each band of EphB4 and ephrin-B2 was normalized to the signal intensity of the band of β-actin obtained in the same lane.

#### Flow Cytometry Assays

##### Isolation of Splenocytes

After suppression, the spleen from *N*, CNT (TNBS and DSS), and EphB4 (TNBS) mice was removed, mechanically dispersed through a 100-μm cell strainer, and washed with PBS containing 0.6 mM Ethylenediaminetetraacetic acid (EDTA) (PBS-EDTA). The cellular suspension was then centrifuged at 1,500 RCF for 10 min at 4°C, the pellet re-suspended in PBS-EDTA, incubated with 2 mL of NH_4_Cl lysis buffer (0.15 M NH_4_Cl, 1 mM KHCO_3_, 0.1 mM EDTA in distilled water) for 5 min, in the dark, to provoke erythrocytes lysis and centrifuged at 1,500 RCF for 10 min at 4°C. Then, pellets were washed with PBS-EDTA and resuspended in 5-mL cell staining buffer (PBS containing 0.5% fetal calf serum (FCS) and 0.1% sodium azide). Finally, the cellular suspension was stained with fluorescent antibodies (Kruisbeek et al., 2001).

##### Isolation of Mesenteric Lymph Nodes (MLN)

After suppression, harvesting of the whole MLN chain located in the mesentery of proximal colon was performed in *N*, CNT (TNBS and DSS) and EphB4 (TNBS) mice. The explanted tissue was rinsed with PBS, vascular and adipose tissues were removed to isolate MLN, mechanically dispersed through a 100-μm cell-strainer, and washed with Hank’s Balanced Salt Solution (HBSS) containing 5% FCS. The obtained suspension was centrifuged at 1,500 RCF for 10 min at 4°C, the pellet was washed with HBSS containing 5% FCS and re-suspended in 3-mL cell staining buffer. Finally, the cellular suspension was stained with fluorescent antibodies.

##### Immunofluorescent Staining

Prior to staining with antibodies, 200 µL of cellular suspension was incubated with IgG1-Fc (1 µg/10^6^ cells) for 10 min in the dark at 4°C to block non-specific binding sites for antibodies. The following antibodies were used for fluorescent staining: Phycoerythrin-Cyanine 5 (PE-Cy5) conjugated anti-mouse CD3ε (0.25 µg/10^6^ cells, catalog number 15-0031, lot number B226301), fluorescein isothiocyanate (FITC) anti-mouse CD4 (0.25 µg/10^6^ cells, catalog number 100406, lot number B210488), PE anti-mouse CD8a (0.25 µg/10^6^ cells, catalog number 100708, lot number B190687). Cells were incubated with antibodies for 1 h in the dark at 4°C, washed with PBS to remove excessive antibody, and suspended in cell staining buffer to perform flow cytometry analysis. The viability of the cellular suspension was determined through propidium iodide (PI) staining, a membrane impermeable fluorescent dye, excluded by viable cells, that binds to DNA emitting red fluorescence, thus resulting as a suitable marker for dead cells. Cells were incubated with PI 10 µg/mL for 1 min in the dark, at room temperature, and immediately subjected to flow cytometry analysis. Only PI^-ve^ cells were included in the analysis.

Samples were analyzed using InCyte^™^ software (Merck Millipore, Darmstadt, Germany). Cell populations were defined as follows: lymphocytes gated in the Forward Scatter (FSC)-Side Scatter (SSC) plot (FSC low: SSC low); T lymphocytes (CD3^+^ lymphocytes); CD4^+^ helper T lymphocytes (CD3^+^CD4^+^CD8^−^ lymphocytes); CD8^+^cytotoxic T lymphocytes (CD3^+^CD8^+^CD4^−^ lymphocytes). Percentages were reported to the total number of splenocytes or MLN cells of each mouse to calculate the number of cells per population.

### *In Vitro* Assays

#### Mononuclear Cells Culture

Spleens were removed from N mice, and splenocytes were isolated as previously described. Splenocytes suspensions were subjected to 40% to 80% Percoll^®^ (GE-Healthcare, Chicago, IL, USA) density gradient centrifugation. Cells were spun at 1000 RCF for 20 min at 20°C, and mononuclear cells, comprising lymphocytes and monocytes, were collected from the 40% to 80% interface, washed with RPMI-1640 (EuroClone, Pero, MI, Italy), and resuspended in medium containing heat-inactivated 10% fetal bovine serum (FBS) (Gibco, Carlsbad, CA, USA) (Kruisbeek, 2001). Before being cultured, the percentage of splenic mononuclear cells represented by CD3^+^ cells was assessed by flow cytometry as previously described (see Immunofluorescent Staining).

Cells were then plated at 1.0*10^6^ cells/mL in 24-well plates and cultured for 24 h at 37°C in a humidified atmosphere with 5% CO_2_ in the absence or presence of EphB4 10 to 100 ng/mL or cyclosporin A (CsA) 1 µg/mL, used as positive control. Ionomycin (500 ng/mL) and phorbol 12-myristate 13-acetate (PMA) (50 ng/mL) were added to the culture media in the final 4 h of incubation to stimulate cells (Li et al., 2015).

#### Mononuclear Cells Viability by Trypan Blue Exclusion

Cell viability was assessed using a hemacytometer and the trypan blue assay by adding 100 ml of cell suspension (5.0*10^6^ cells/mL) to 900 ml of 0.4% trypan blue solution (Sigma). Ten microliters of each suspension were analyzed in a hemacytometer. Live and dead cells were enumerated under a light microscope. Percent viability was assessed at the time of isolation and after 24 h of incubation with vehicle, EphB4 10 to 100 ng/mL or CsA 1 µg/mL in the absence or presence of ionomycin and PMA (Klein et al., 2006).

#### Determination of TNF-α Levels in Cultured Mononuclear Cells

At the end of the incubation period with the test compounds and in the absence or presence of ionomycin and PMA, the culture media were frozen at −80°C until cytokine measurement. TNF-α levels were determined in duplicate in 100 µL samples, using a commercially available ELISA kit (Mouse TNF alpha ELISA Ready-SET-Go!, eBioscience^™^, San Diego, CA). The absorbance was measured spectrophotometrically at 450 nm (TECAN Sunrise^™^ powered by Magellan^™^ data analysis software, Mannedorf, Switzerland). The assays sensitivity was 8 pg/mL.

#### ELISA Binding Assays

ELISA assays were performed as previously described (Giorgio et al., 2019). Briefly, 96-well ELISA high binding plates (Costar, Corning, New York, NY, USA 9018) were incubated overnight at 4°C with 100 µL/well of 1 µg/mL EphB1-Fc (R&D systems, Minneapolis, MN, USA, 1596-B1) or EphB4-Fc (R&D systems, Minneapolis, MN, USA, 446-B4) diluted in sterile phosphate buffered saline (PBS—0.2 g/L KCl, 8.0 g/L NaCl, 0.2 g/L KH_2_PO_4_, 1.15 g/L Na_2_HPO_4_, pH 7.4). The day after, wells were washed with washing buffer (PBS + 0.05% tween20, pH 7.4) and blocked with 300 µl/well with blocking solution (PBS + 0.5% BSA PanReac Applichem, Darmstadt, Germany A1391,0050) for 1 h at 37°C. EphB4 (His Tag) (Sino Biological Inc., Beijing, China 50582-M08H) was first incubated at 1 to 10,000 ng/ml with biotinylated ephrin-B1-Fc or ephrin-B2-Fc for 30 min at room temperature and then added to the wells at 37°C for 1 h. Finally, wells were washed and incubated with 100 µL/well Streptavidin-HRP (Sigma-Aldrich, Milan, Italy, S5512) for 20 min at room temperature, washed again and incubated at room temperature with 100 µl/well 0.1 g/L tetra-methylbenzidine (Sigma-Aldrich, Milan, Italy, 860336) reconstituted in stable peroxide buffer (11.3 g/L citric acid, 9.7 g/L sodium phosphate, pH 5.0), and 0.02% H_2_O_2_ (30% w/w in water), added immediately before use. The reaction was stopped with 3N HCl 100 µL/well, and the absorbance was measured using an ELISA plate reader (Sunrise, TECAN, Switzerland) at 450 nm.

### Statistics

All data were presented as means ± SEM. Comparison among experimental groups was made using analysis of variance (one-way or two-way ANOVA) followed by Bonferroni’s post-test, when *P* < 0.05, chosen as level of statistical significance, was achieved. Non-parametric Kruskal–Wallis analysis, followed by Dunn’s post-test, was applied for statistical comparison of MS. All analyses were performed using Prism 4 software (GraphPad Software Inc. San Diego, CA, USA).

### Drugs, Antibodies, and Reagents

Recombinant rat EphB1-Fc chimera and recombinant mouse ephrinB1-Fc chimera were purchased from R&D systems^™^ (Minneapolis, MN), mouse EphB4 (His Tag) from Sino Biological Inc.^™^ (Beijing, China). FITC anti-mouse CD4, PE anti-mouse CD8, and propidium iodide were purchased from BioLegend^™^ (San Diego, CA, USA), PE-Cy5 anti-mouse CD3 from Affymetrix eBioscience^™^ (San Diego, CA) and IgG1-Fc from Millipore^™^ (Merck, Darmstadt, Germany). DSS was purchased from MP Biomedicals^®^ (Germany). TNBS, cyclosporine A, ethanol, HTAB, hydrogen peroxide, and all the other reagents were purchased from Sigma Aldrich^™^ (St. Louis, MO, USA). Drugs were dissolved in saline solution (recombinant proteins) or carboxymethylcellulose 0.5% (CsA) the day of the experiment.

## Results

### Instillation of TNBS Evoked a Severe Inflammatory Response

**Figure 1:**
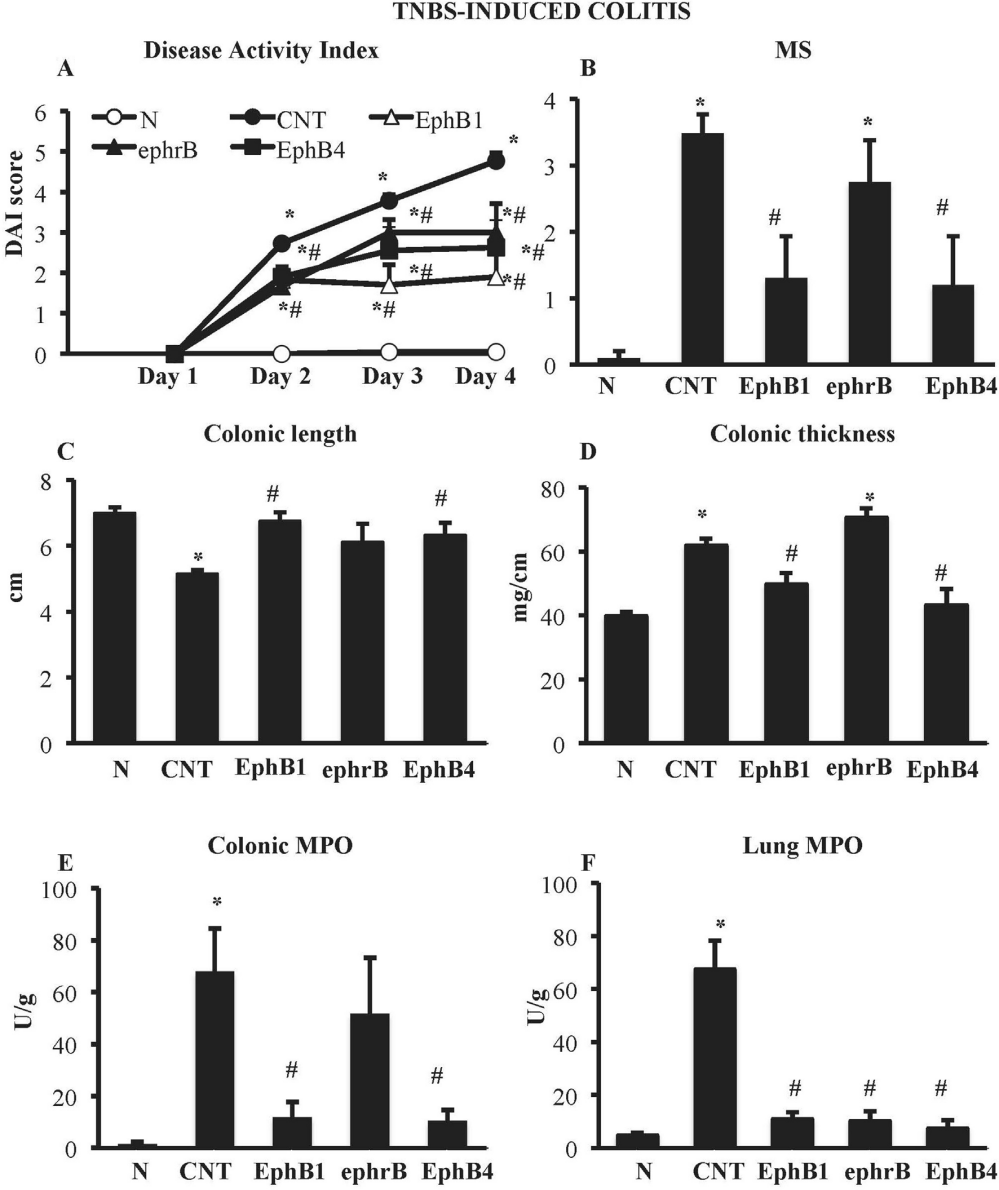
Effects of EphB/ephrinB ligands on 2,4,6-trinitrobenzenesulfonic acid (TNBS)-induced inflammatory responses. Disease Activity Index **(A)**, macroscopic score **(B)**, colonic length **(C)**, colonic thickness **(D)**, colonic myeloperoxidase (MPO) **(E)**, and lung MPO **(F)** activity assessed in vehicle-treated normal mice (N) and in TNBS-treated mice administered with vehicle (CNT), EphB1-Fc 30 µg/kg (EphB1), ephrin-B1-Fc 17 µg/kg (ephrB), EphB4 20 µg/kg (*n* = 5–12 independent values per group). *P < 0.05 vs. N mice; ^#^ *P* < 0.05 vs. CNT mice; one-way ANOVA followed by Bonferroni’s post-test.

**Figure 2:**
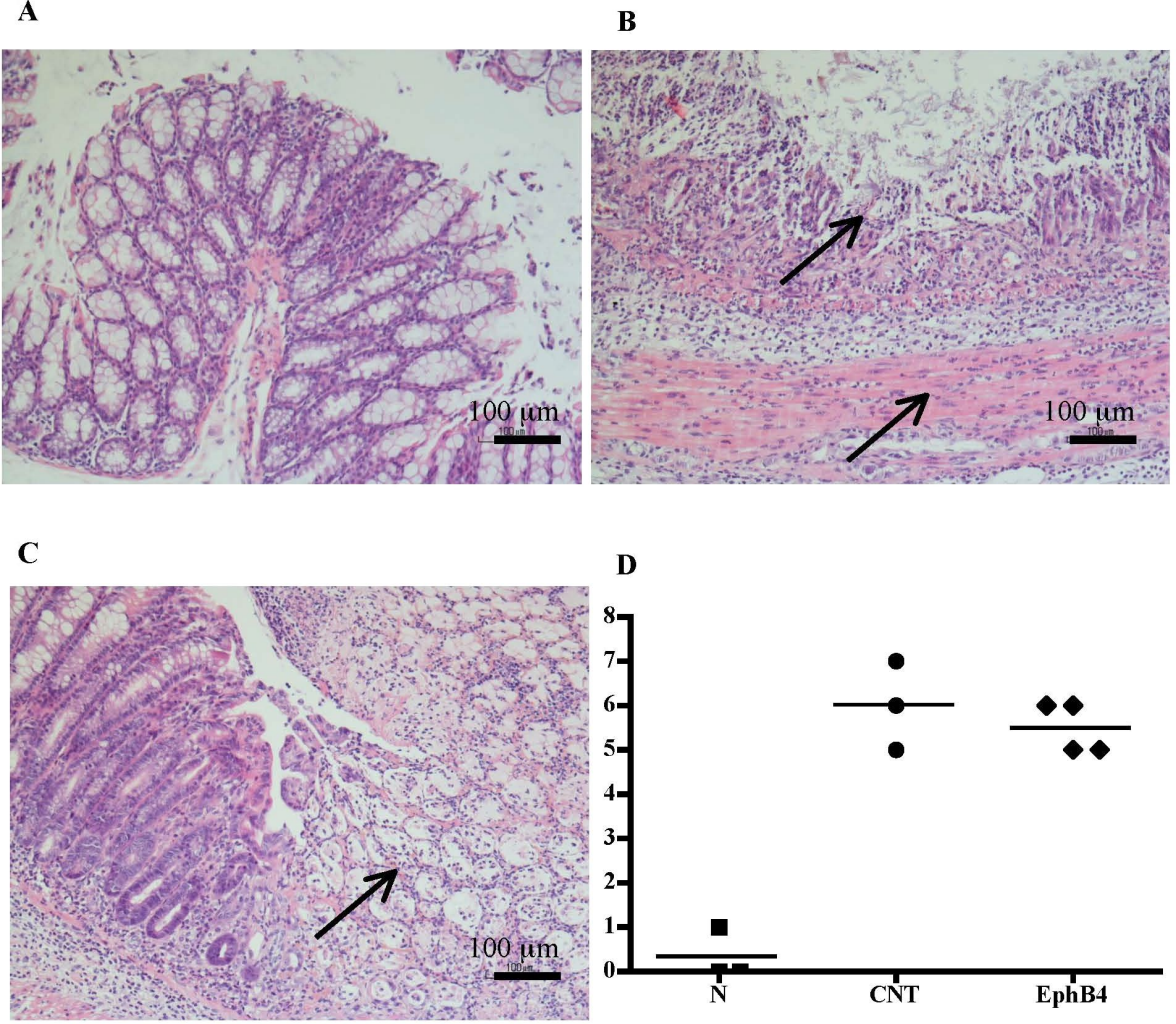
Histology. Representative hematoxylin–eosin-stained sections of colonic specimens harvested from vehicle-treated normal mice **(A)** and from TNBS-treated mice administered with vehicle **(B)** or EphB4 20 µg/kg **(C)**. TNBS colonic instillation caused mucosal necrosis, neutrophils infiltration, and submucosal edema (indicated by arrows) in vehicle-treated animals not attenuated in EphB4-treated mice **(C)**. Panel **D** represents histological damage scoring of colonic sections obtained from vehicle-treated normal mice (▪), CNT mice (•), or EphB4-treated colitic mice (◆) (horizontal bar at the mean value).

Compared with the N group, CNT mice showed a significantly higher DAI value (**Figure 1A**), caused by both body weight loss and softening of stools. Inoculation of the haptenating agent induced also a remarkable colonic shortening and thickening and injury of the mucosa, evaluated as MS (**Figure 1B–D**). Colonic and pulmonary myeloperoxidase activity was severely augmented upon colitis induction (**Figure 1E and F**) indicating a conspicuous recruitment of granulocytes within tissues. As a result, also colonic IL-1β levels were increased in CNT mice compared with the *N* group (*N*: 11.7 ± 3.0 pg/mg proteins vs. CNT: 221.5 ± 45.0 pg/mg proteins; *P* < 0.05). Microscopic histological analysis showed extensive mucosal necrosis and conspicuous granulocytes infiltration both in the lamina propria and in the submucosa in TNBS-exposed mice (**Figure 2B**; histological score: 6.0 ± 0.6; **Figure 2D**) compared with normal mice (**Figure 2A**; histological score: 0.3 ± 0; **Figure 2D**).

### EphB4 and EphB1-Fc Dampened TNBS-Induced Inflammatory Responses

Colitic mice were subjected to repeated s.c. treatment with monomeric EphB4 protein or with Fc-conjugated chimeric proteins, containing equimolar doses of either ephrin-B1 or EphB1 ectodomains. EphB4 protein 20 µg/kg elicited protective effects against colitis: a marked reduction of DAI and of colonic macroscopic damage, thickness, and shortening occurred. Granulocyte infiltration within colon and lungs was deeply contrasted (**Figure 1**) and, similarly, colonic IL-1β levels were attenuated (87.52 ± 15.16 vs. CNT: 221.49 ± 44.96 pg/mg proteins), although no relevant protection against TNBS-induced intestinal injury emerged from the histological analysis (**Figure 2C**; histological score: 5.3 ± 0.3; **Figure 2D**). When tested at 5 and 10 µg/kg, EphB4 showed dose-dependent effects, being able to counteract colonic shortening and thickening at 10 µg/kg (**Supplementary Figure 1**).

Similar to EphB4, EphB1-Fc 30 µg/kg consistently reduced DAI and MS, counteracted colonic shortening and thickening and dampened the increase of MPO levels in colon and lungs (**Figure 1**). On the other hand, mice treated with equimolar ephrinB1-Fc (17 µg/kg) showed MPO activity lower than controls only in lungs (**Figure 1F**).

Finally, treatment with IgG1-Fc 10 µg/kg, tested as negative control, did not significantly modify any of the inflammatory parameters increased by TNBS enema, albeit for colonic length (**Supplementary Table 1**).

### DSS Administration Elicited a Severe Colitis Not Attenuated by EphB/EphrinB Modulation

**Figure 3:**
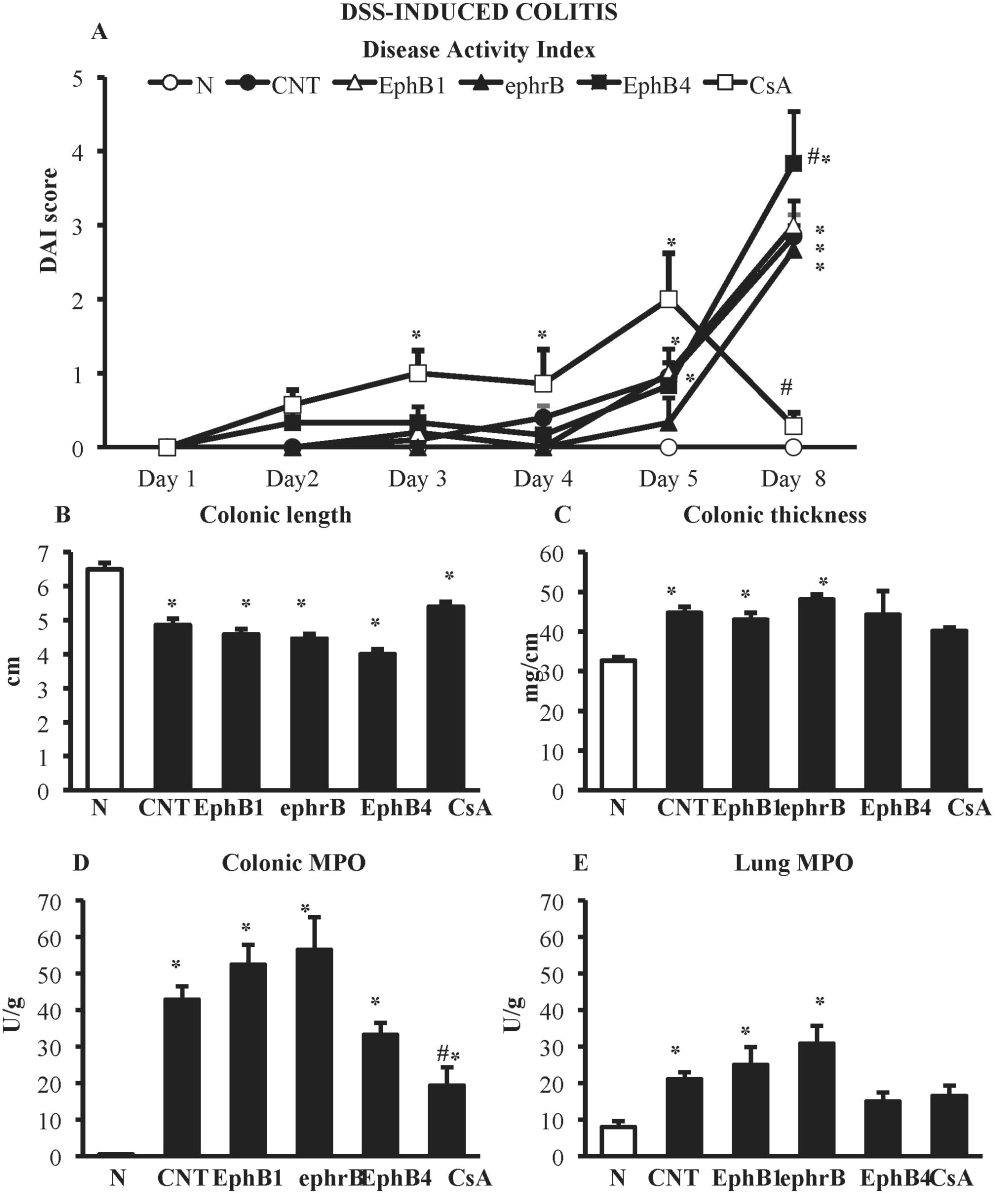
Effects of EphB/ephrinB ligands on dextran sulphate sodium (DSS)-induced inflammatory responses. Disease Activity Index **(A)**, colonic length **(B)**, colonic thickness **(C)**, colonic MPO **(D)** and lung MPO **(E)** activity assessed in vehicle-treated normal mice (N) and in DSS-treated mice administered with vehicle (CNT), EphB1-Fc 30 µg/kg (EphB1), ephrin-B1-Fc 17 µg/kg (ephrB), EphB4 20 µg/kg (*n* = 5–12 independent values per group). *P < 0.05 vs. N mice; ^#^ *P* < 0.05 vs. CNT mice; one-way ANOVA followed by Bonferroni’s post-test.

DSS administration caused severe colitis characterized by worsening of DAI score and of the local parameters of inflammation and damage: colon length was markedly decreased, whereas colon thickness and colonic myeloperoxidase activity were significantly augmented. On the contrary, no mucosal lesions, assessed as MS, could be detected following DSS treatment. A huge infiltration of neutrophils was triggered in the lungs, indicating a strong systemic inflammation (**Figure 3**).

In the DSS model, neither the administration of Fc-conjugated agonists, EphB1-Fc, and ephrinB1-Fc nor the use of monomeric protein EphB4 were effective in controlling the burden of DSS-induced damage; on the contrary, interference with EphB/ephrin-B signaling even worsened mice general clinical conditions (**Figure 3**). Only treatment with immunosuppressant cyclosporine A 25-mg/kg i.p., tested as positive control, was able to reduce DAI score at day 8 (CsA 0.3 ± 0.2 vs. CNT 2.9 ± 0.3, *P* < 0.05) and colonic neutrophils infiltration (MPO activity: CsA 19.3 ± 5.0 U/g vs. CNT 42.9 ± 3.7 U/g, *P* < 0.05) with respect to control values, leaving unmodified the other responses (**Figure 3**).

### Treatment With EphB4 Did Not Influence Colonic EphB4 and Ephrin-B2 Genes Expression

**Figure 4:**
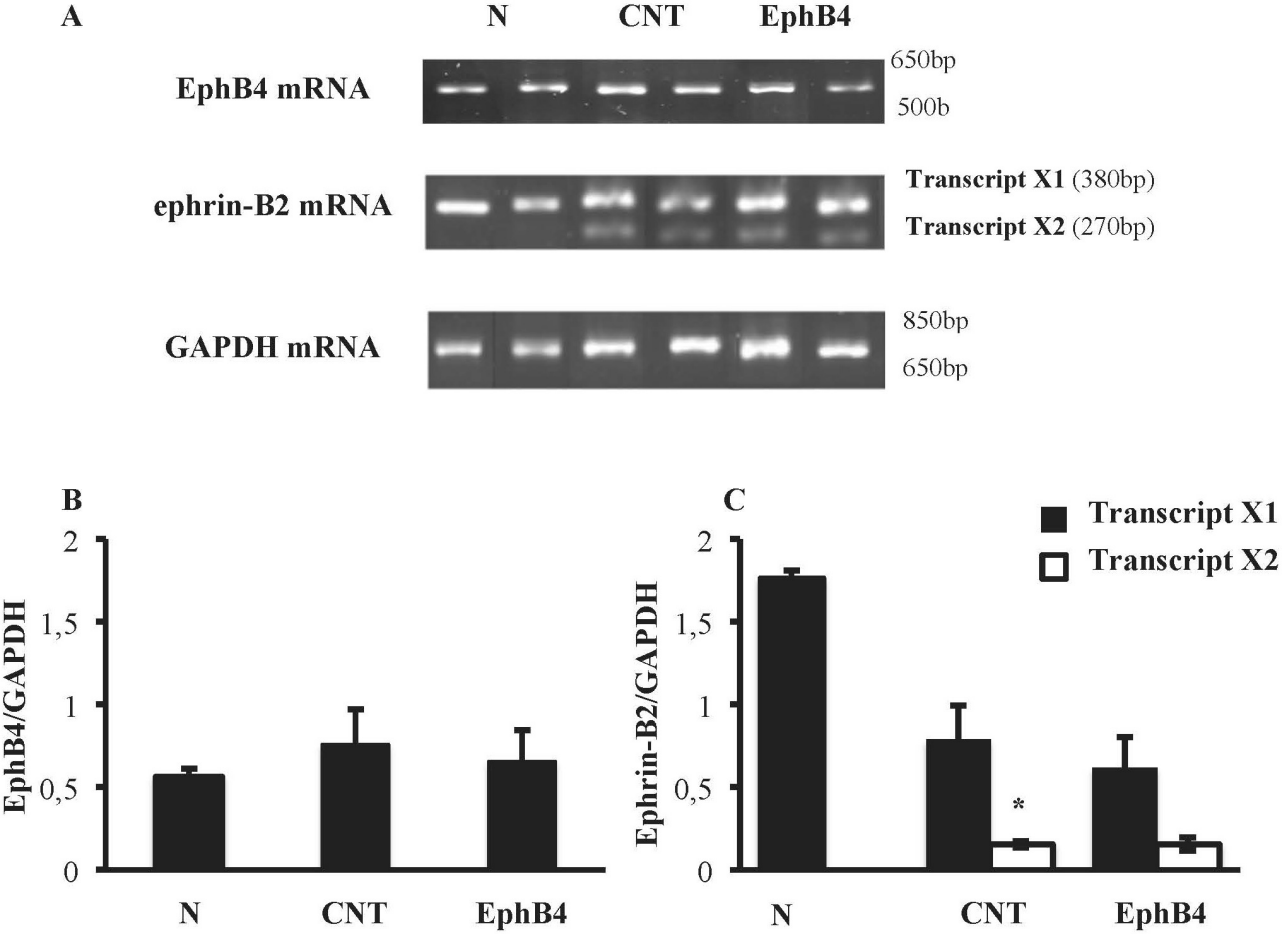
Colonic levels of EphB4 and ephrin-B2 mRNA. **(A)** Representative agarose gels showing mRNA levels of EphB4, ephrin-B2 and GAPDH from the colon of vehicle-treated normal mice (N; *n* = 7) and of TNBS-treated mice administered with vehicle (CNT; *n* = 4) or EphB4 20 µg/kg (EphB; *n* = 5). Histograms represent the quantification of EphB4 **(B)** and ephrin-B2 **(C)** mRNA levels normalized to GAPDH amplification products. Transcript X1 (black bars) and transcript X2 (white bars), encoded by ephrin-B2 gene, were detected and represented **(C)**. Data are shown as mean ± SEM. *P < 0.05 vs. N mice, one-way ANOVA followed by Bonferroni’s post-test.

**Figure 5:**
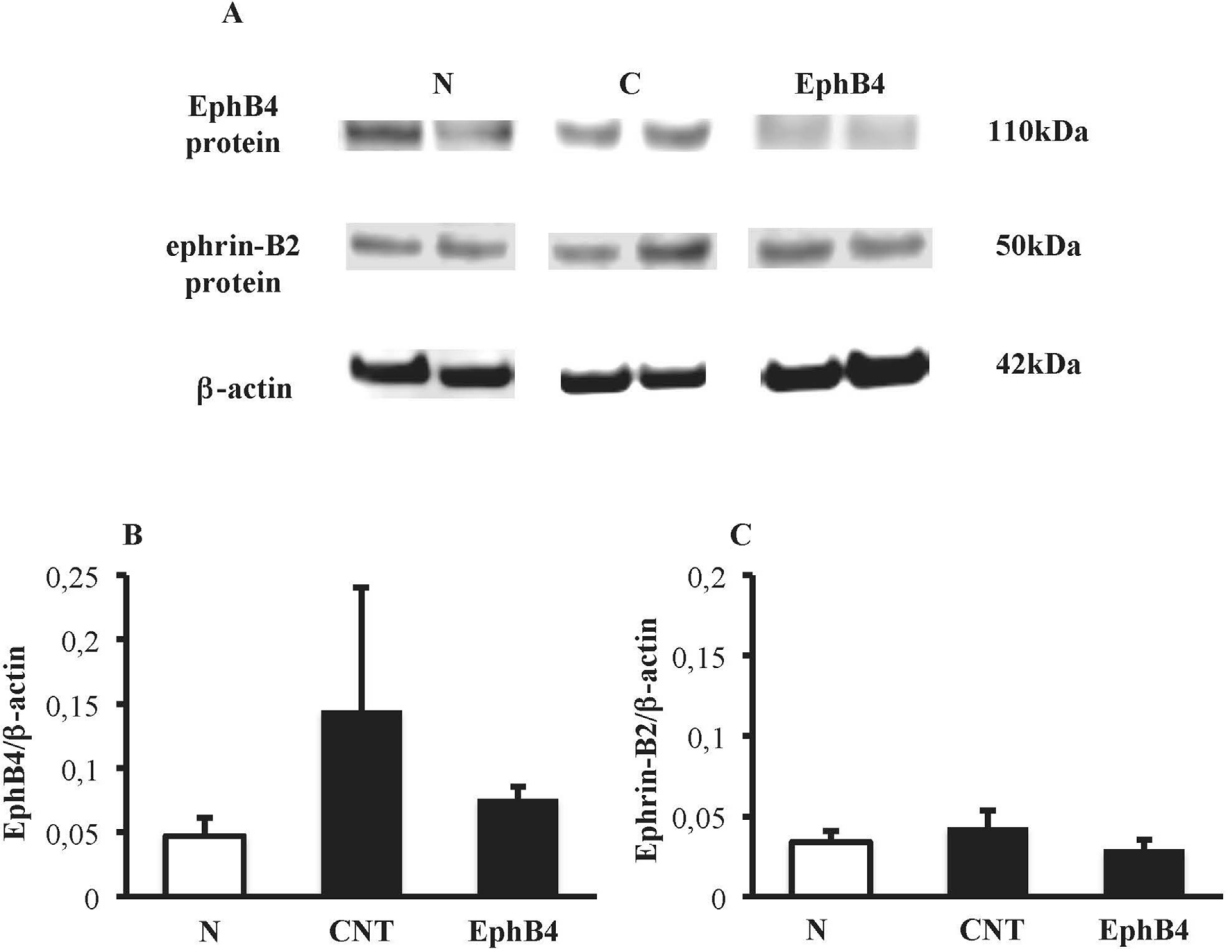
Colonic levels of EphB4 and ephrin-B2 proteins. **(A)** Representative western blots showing the expression of EphB4, ephrin-B2 and β-actin proteins in lysates, obtained from the colon of vehicle-treated normal mice (N) and in TNBS-treated mice administered with vehicle (CNT) or EphB4 20 µg/kg (EphB4). Histograms represent the densitometric analysis of EphB4 **(B)** and ephrin-B2 **(C)** blots, normalized to β-actin (*n* = 3–4 per group).

Colonic tissues of N mice showed single bands of EphB4 and ephrin-B2 (transcript X1) mRNA transcripts at 550 and 380 bp, respectively. In CNT mice exposed to TNBS, the levels of EphB4 mRNA were unchanged with respect to N mice; as regard ephrin-B2, a second mRNA isoform appeared at 270 bp (transcript X2), not evident in N mice (*P* < 0.05 vs. N), whereas the levels of transcript X1 were slightly decreased. Sanger sequencing of ephrin-B2 cDNA (Microsynth AG, Switzerland) confirmed that the two bands corresponded to splice variants of ephrin-B2 gene differing by 93 bp. Treatment with EphB4 did not significantly change EphB4 or ephrin-B2 gene expression with respect to CNT group (**Figure 4**). As regard EphB4 and ephrin-B2 proteins expression in the colon, only single bands could be detected through Western blot analysis, and signal intensity was comparable in all groups (**Figure 5**).

### EphB4 Affects TNBS-Induced Changes in T-Cell Profile

**Figure 6:**
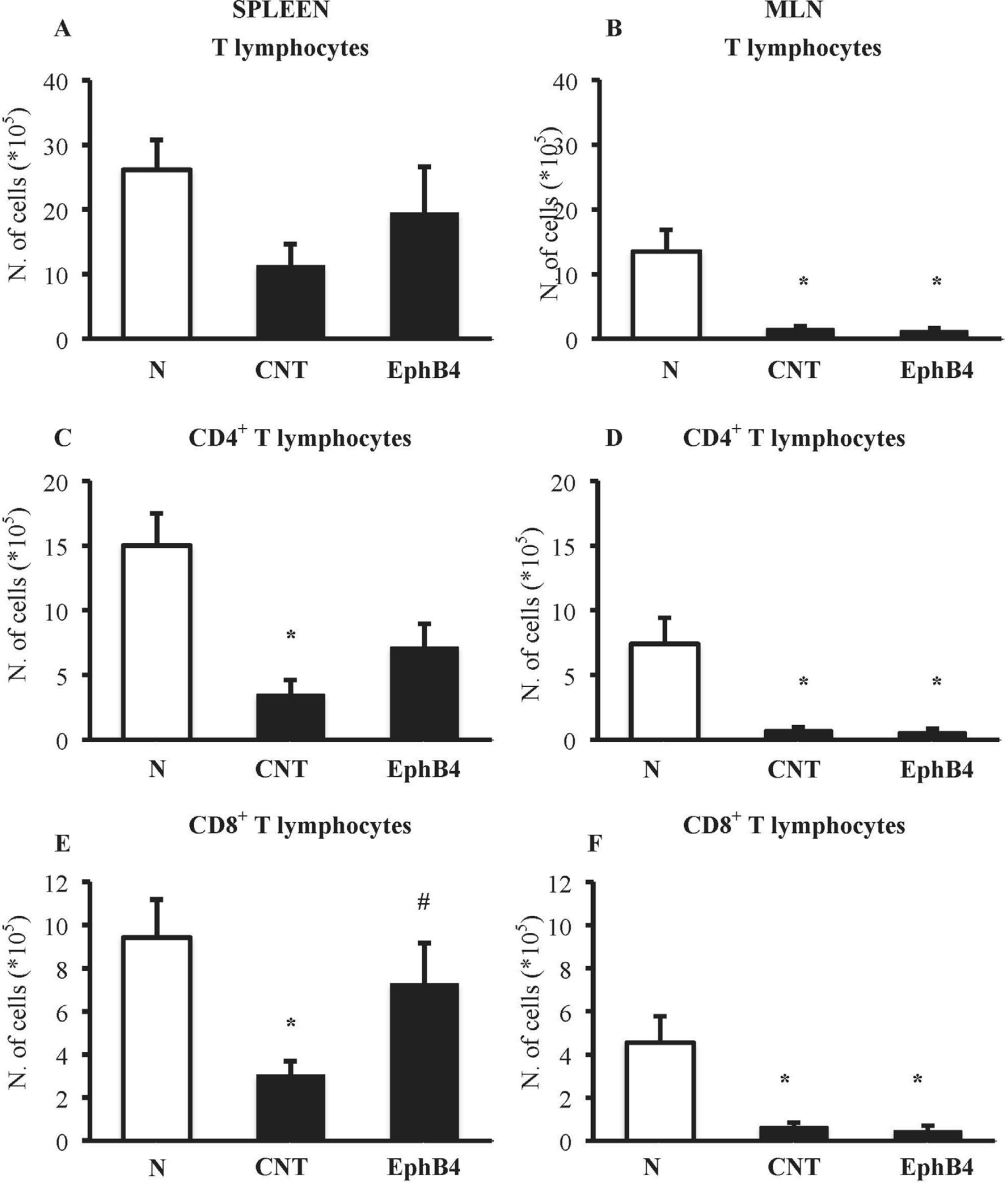
EphB4 attenuated TNBS-induced changes in spleen T-cell profile. Number of T lymphocytes (CD3^+^) **(A, B)**, CD4^+^ **(C, D)**, and CD8^+^ **(E, F)** T lymphocytes in the spleen **(A, C, E)** and in MLN **(B, D, F)** excised from vehicle-treated normal mice (N) and TNBS-treated mice administered with vehicle (CNT) or EphB4 20 µg/kg (EphB4) (*n* = 5–9 independent values per group). *P < 0.05 vs. N mice; ^#^ *P* < 0.05 vs. CNT mice, one-way ANOVA followed by Bonferroni’s post-test.

The phenotypic characterization of splenic and MLN T-cells was performed in N and CNT mice: TNBS instillation decreased CD4^+^ and CD8^+^ T cells in the spleen and in MLN (**Figure 6**). Interestingly, when we tested the effect of EphB4 treatment, the monomeric protein increased the number of CD4^+^ and especially of CD8^+^ T cells in the spleen, leaving unmodified MLN populations (**Figure 6**).

On the contrary, DSS exposure did not remarkably affect lymphocytes populations, the number of CD4^+^ and CD8^+^ T cells being essentially unaltered in either lymphoid tissues (**Supplementary Figure 2**).

### EphB4 Promoted TNFα Release From Splenic Mononuclear Cells

**Figure 7:**
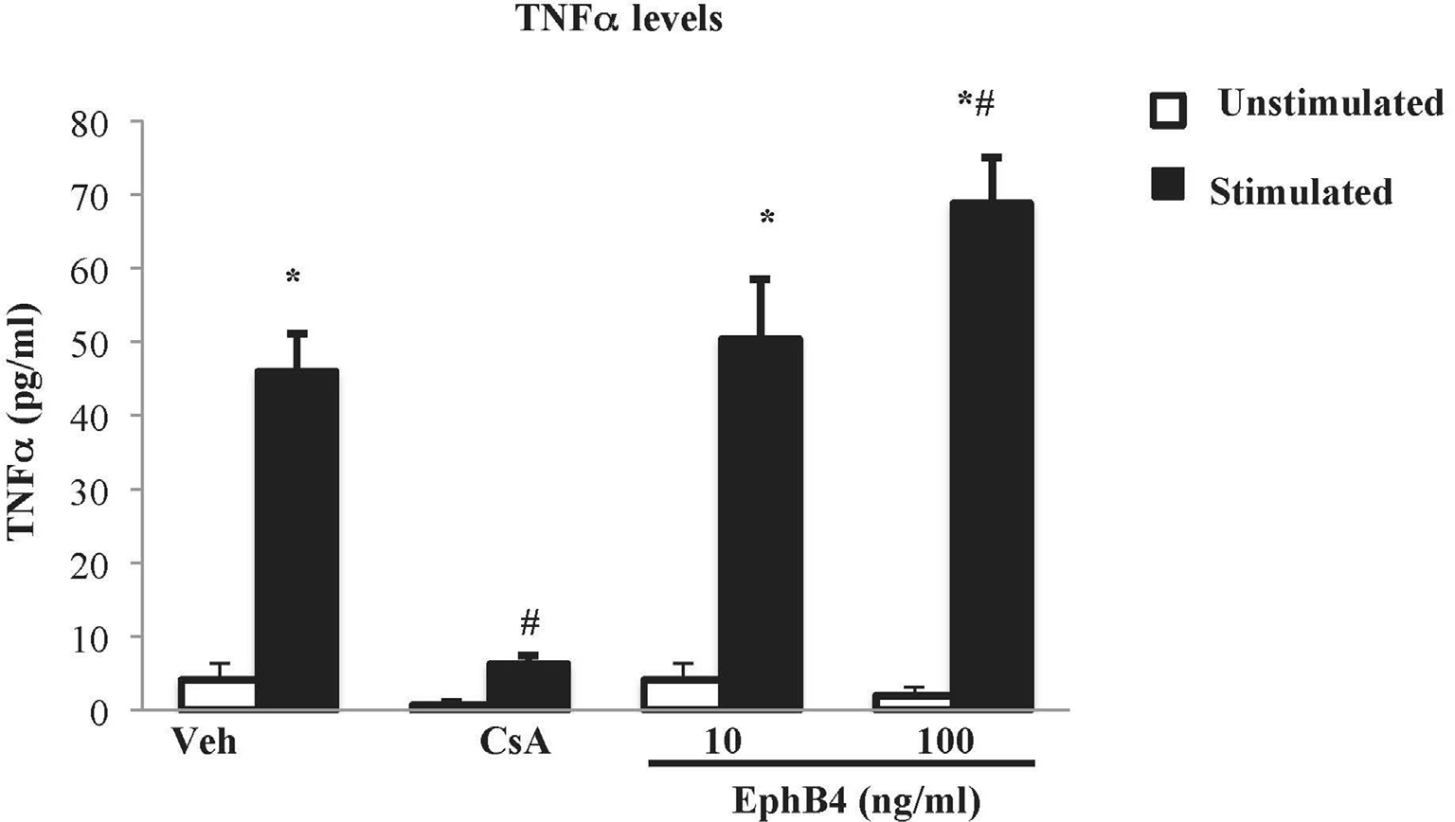
Effects of EphB4 on splenic mononuclear cells activation. TNFα production by cultured splenic mononuclear cells incubated with vehicle (Veh), cyclosporine A 1 µg/ml (CsA) or EphB4 (10–100 ng/ml) (EphB4) in the presence (black bars) or absence (white bars) of PMA (50 ng/mL) and ionomycin (500 ng/mL) (*n* = 4–10 independent values per group). *P < 0.05 vs. unstimulated; ^#^ *P* < 0.05 vs. corresponding vehicle; two-way ANOVA followed by Bonferroni’s post-test.

The effects of EphB4 were also investigated *in vitro* on splenic mononuclear cell culture activation. Flow cytometry confirmed that around half of the isolated splenocytes set into culture were represented by CD3^+^ lymphocytes (48.7% ± 6.6%). Then, their viability was ascertained by trypan blue exclusion assay: in case the percentage of viable cells was higher than 95%, cells were cultured, incubated with the test compounds or vehicle, stimulated by PMA and ionomycin, and TNF-α levels were determined in the cell culture media. After 24 h of incubation with vehicle, the vitality was maintained (**Supplementary Table 2**) and the cytokine levels were significantly augmented in stimulated cells compared with the unstimulated conditions (**Figure 7**). Cyclosporine A 1 µg/ml was able to abolish TNF-α release induced by PMA and ionomycin while maintaining lymphocytes viability. As regard the monomeric protein, at the higher tested concentration, EphB4 enhanced the increase of TNF-α levels raised by the stimulants, without affecting cells vitality (**Figure 7** and **Supplementary Table 2**).

### Binding Affinity of Monomeric EphB4 to Ephrin-B1 and Ephrin-B2

**Figure 8:**
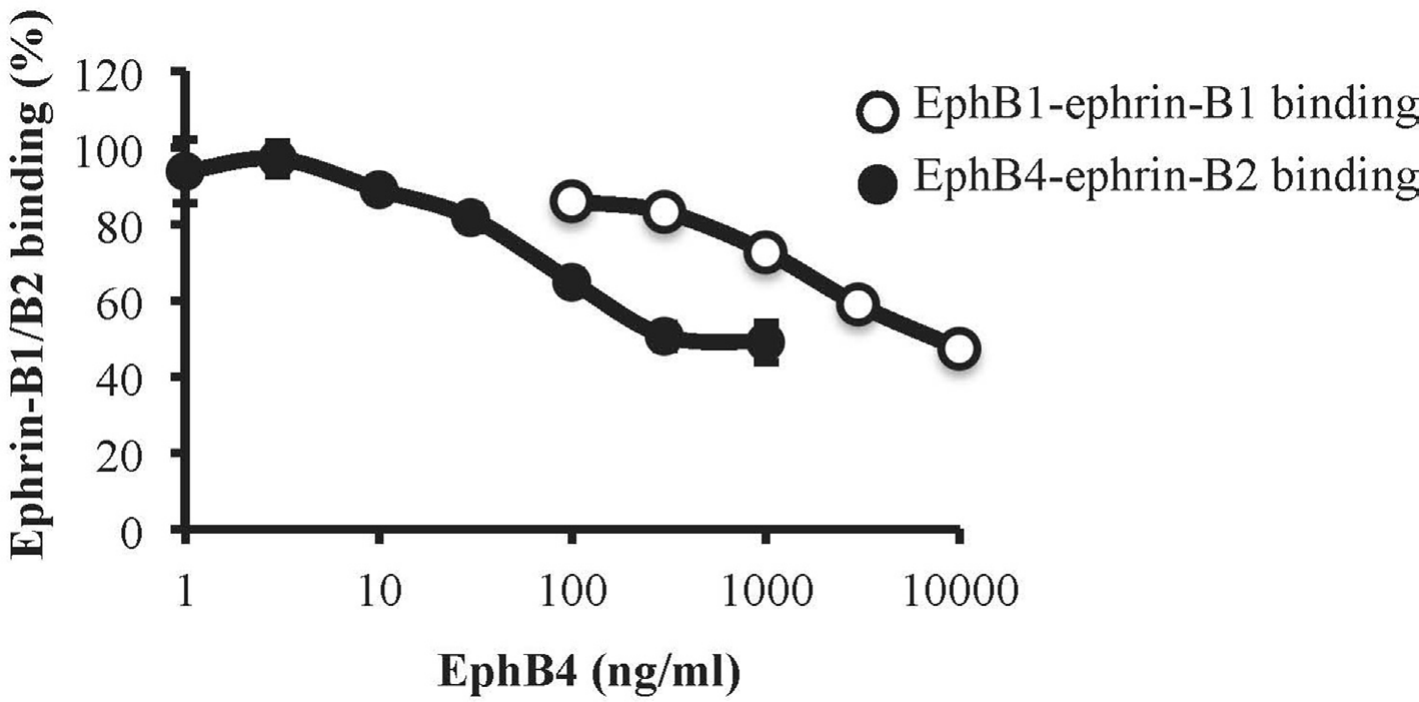
Binding affinity of monomeric EphB4 to ephrin-B1 and ephrin-B2. EphB4 concentration-dependently displaced the binding of biotinylated ephrin-B1-Fc from immobilized EphB1-Fc (white circles) or the binding of biotinylated ephrin-B2-Fc from immobilized EphB4-Fc (black circles) (*n* = 3 independent experiments).

To assess the affinity profile of EphB4 towards ephrin-B1 and ephrin-B2 proteins, we immobilized the EphB1-Fc and EphB4-Fc ectodomains on ELISA plates and then promoted the binding of biotinylated ephrin-B1-Fc and ephrin-B2-Fc, respectively, according to a previously published protocol (Giorgio et al., 2019). Monomeric EphB4 was able to concentration-dependently displace both ephrin-B1-Fc/EphB1-Fc and ephrin-B2-Fc/EphB4-Fc binding, but antagonized ephrin-B2-Fc/EphB4-Fc binding at concentrations more than 20 times lower than those interfering with ephrin-B1-Fc/EphB1-Fc interaction (**Figure 8**).

## Discussion

The multiplicity of cell targets, involved in inflammatory responses, affected by EphB/ephrin-B signaling pathways (Kania and Klein, 2016) and the documented up-regulation of ephrin-B2 mRNA levels in gut mucosal lesions of CD patients (Hafner et al., 2005) prompted us to focus our attention on the possible involvement of EphB/ephrin-B system in intestinal inflammatory disorders. To this end we applied two murine models of IBD characterized by complementary pathogenetic mechanisms: the TNBS model, primarily driven by Th1-mediated immune responses, and the DSS one, mainly triggered by innate immunity responses and useful to highlight the efficacy of agents able to re-establish the integrity of the epithelium after injury (Kiesler et al., 2015). In our experimental conditions, both TNBS and DSS acute exposures produced inflammatory responses of remarkable intensity, with gradual impairment of general physical conditions, colonic mucosal injury, shortening and thickening, and abundant recruitment of colonic and pulmonary neutrophils. However, despite the analogies, the pharmacological manipulation of EphB/ephrin-B system produced distinct outcomes in the two models. Treatment with recombinant proteins was ineffective in attenuating DSS-induced colitis, only cyclosporine being able to mitigate clinical severity and colonic neutrophil recruitment; at variance, blockade of EphB/ephrin-B signaling markedly lowered both the local and systemic inflammatory responses elicited by TNBS, improving health conditions and counteracting colonic changes, leukocytes enrolment, and, accordingly IL-1β production. Specifically, overlapping results were obtained, on one side, by interfering with both forward and reverse signaling through monomeric EphB4 and, on the other hand, by blocking forward signaling and activating the reverse one through EphB1-Fc. Accordingly, we could speculate that endogenous EphB/ephrin-B forward signaling plays a significant role in TNBS-induced inflammation and that its antagonism could represent a novel, promising approach to control the hapten-induced flogosis.

Given the increased levels of ephrin-B2 mRNA in the gut mucosal lesions of CD patients (Hafner et al., 2005), we then asked whether similar changes in the expression of ephrin-B2 ligand and of its preferential binding protein, the EphB4 receptor, occurred in TNBS-induced colitis: as a result, comparable levels of EphB4 mRNA and protein were detected in the colon of normal and colitic mice. As regard ephrin-B2 gene transcripts, the analysis revealed the presence of two bands corresponding to splice variants of ephrin-B2 gene. The existence of an alternate splice isoform of ephrin‐B2, in which a conserved region of 31 amino acid residues was truncated, was demonstrated by Bergemann more than 20 years ago in mouse embryos nervous system (Bergemann et al., 1995); however, to the best of our knowledge, this is the first proof of its presence in mouse inflamed intestinal tissue. It is likely that pro-inflammatory cues may promote the altered expression of this shorter splice variant, as it has been demonstrated for other genes in IBD (Manousou et al., 2008; Matos et al., 2013); to this end, future studies will be pivotal to determine whether similar findings can be evidenced also in the colonic mucosa of other murine models of colitis and of IBD patients and to unravel its specific function. Interestingly, treatment with EphB4 was not able to prevent the generation of the alternative variant, despite its efficacy in counteracting the inflammatory responses in TNBS-induced colitis: this suggests that the anti-inflammatory action modulates down-stream factors apparently not involved in the transcriptional regulation of ephrin-B2 gene. As regard the mature protein, only a single band, presumably corresponding to ephrin-B2 longer product, could be detected through western blotting: technical issues, related to the low sensitivity of our experimental procedures, might explain the difficulty in revealing the protein encoded by the shorter mRNA sequence.

To get a deeper insight into the mechanism of the protective action afforded by EphB/ephrin-B antagonism specifically in TNBS-induced colitis, and given the pivotal role played by T lymphocytes in this model, we focused on adaptive immune cells involvement. Indeed, although the injury to colonic epithelium produced by DSS exposure is the first step leading to recruitment of innate immune cells, TNBS-induced colitis, triggered by haptenization of colonic tissue proteins, is considered primarily based on the activation of CD4^+^ Th1-mediated responses (Kiesler et al., 2015). Accordingly, in our TNBS model, a marked reduction of CD4^+^ and CD8^+^ T lymphocytes was evident both in the spleen and MLN of colitic mice, suggesting the migration of T lymphocytes from secondary lymphoid organs to the site of inflammation, whereas no significant changes could be detected after DSS assumption. The ability of EphB4 to prevent the outflow of T cells from the spleen promoted by TNBS could presumably lead to a lower recruitment of adaptive immune cells, besides neutrophils, into the lamina propria, confirming our hypothesis of a protection involving T-cell modulation. Future investigations will help to unravel the matter.

Finally, we investigated the effects of EphB4 on the production of TNFα by isolated splenic mononuclear cells exogenously stimulated. It is well known that mitogen PMA and ionophore ionomycin stimulate cytokine gene expression in T lymphocytes through Ca^2+^-dependent pathways, bypassing the TCR complex (Truneh et al., 1985; Savignac et al., 2007) and, as confirmed also by our results, in a manner sensitive to cyclosporine A, immunosuppressant drug able to block Ca^2+^-dependent responses (Almawi et al., 1999). Intriguingly, EphB4 was able to potentiate the production of TNFα from activated splenic mononuclear cells at a concentration interacting with ephrin-B2 but not with ephrin-B1. Despite the still under-investigated contribution of Eph/ephrin system to the physiological regulation of immune responses, the ability of monomeric EphB4 to modulate mononuclear cells activation may reflect the role played by EphB4/ephrin-B2 couple in the regulation of lymphocytes activity: in particular, both stimulation and inhibition of T lymphocytes proliferation and pro-inflammatory cytokines release have been described for ephrin-B2/EphB forward signaling following anti-CD3 stimulation (Yu et al., 2003; Kawano et al., 2012). These observations led us to speculate that, in our experimental conditions, the increase in *in vitro* TNFα levels promoted by EphB4 is a possible consequence of the blockade of EphB forward signaling in splenic mononuclear cells and a result only apparently conflicting with its protective action in TNBS-induced colitis. Indeed, inside the multiform scenario of the biological actions of TNFα, both pathological and homeostatic responses can be elicited by this cytokine in the gastrointestinal tract. If, on one side, anti-TNFα agents represent the mainstay in IBD therapy, also paradoxical GI adverse events for drugs neutralizing TNFα as well as the improvement of mucosal healing by TNFα during colitis have been reported over the past years (Bradford et al., 2017), reinforcing the notion of the Janus-faced role of this cytokine in intestinal inflammation (Dubé et al., 2015). Accordingly, the promotion of lymphocytes activation and of TNFα production by EphB4/ephrin-B2 blockade may contribute to balance the immune surveillance, controlling the flogistic cascade elicited by TNBS.

**Figure 9:**
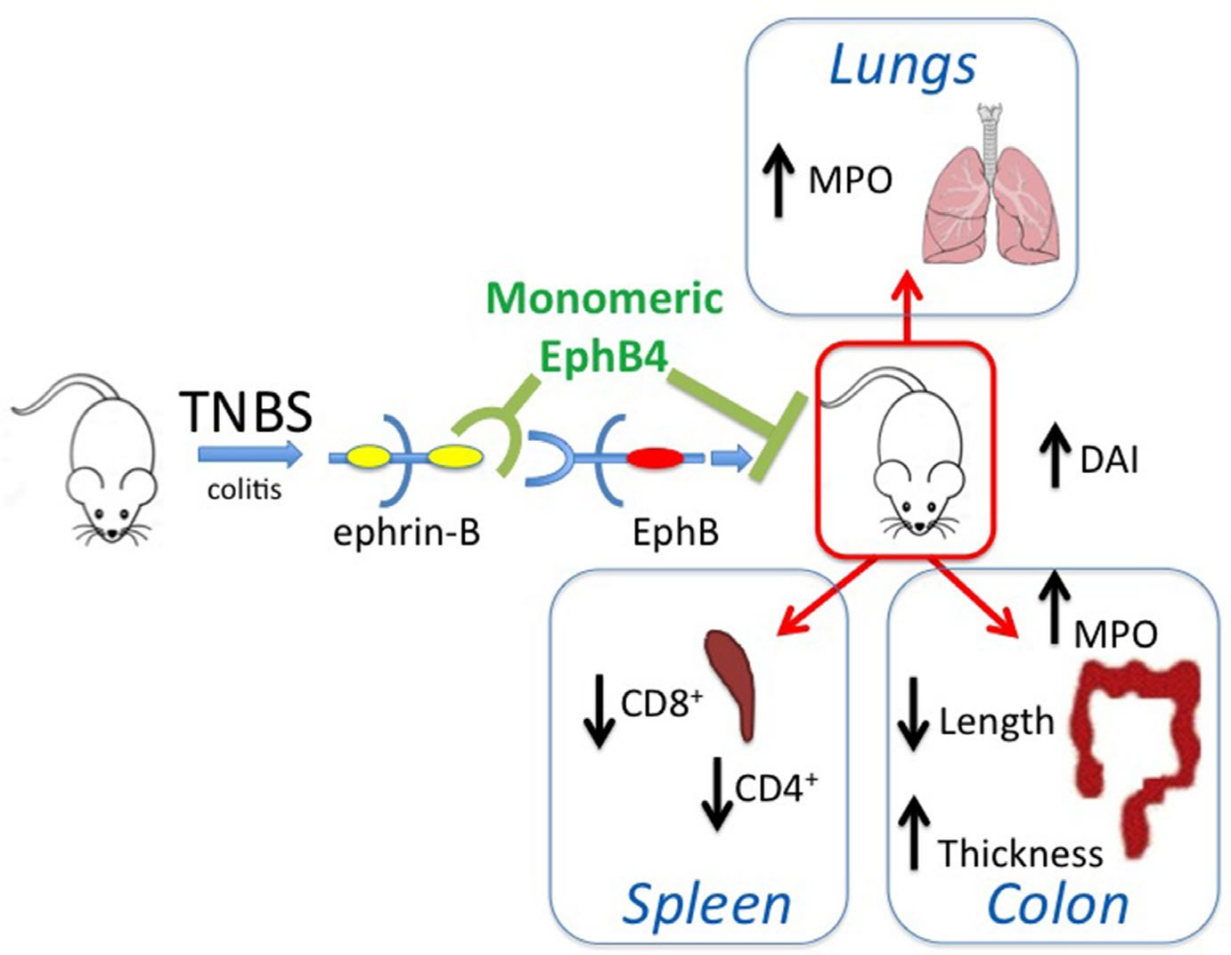
Graphical summary. The scheme describes the main findings of the manuscript and represents the ability of monomeric protein EphB4 to counteract the TNBS-induced inflammatory responses by interfering with EphB–ephrin-B interaction.

On the whole, the collected results, schematically represented in **Figure 9**, point to a potential role of the EphB/ephrin-B system as pharmacological target in intestinal inflammatory disorders and suggest that the therapeutic efficacy achieved by its blockade seemingly works through the modulation of immune responses and independent of changes at the transcriptional and translational level of EphB4 and ephrin-B2 genes in intestinal tissues.

## Author’s Note

Preliminary data were presented at Digestive Disease Week 2018, June 2–5 2018, Washington (DC) (USA).

## Data Availability Statement

All datasets generated for this study are included in the manuscript and the supplementary files.

## Ethics Statement

Animal experiments were performed according to the guidelines for the use and care of laboratory animals, and they were authorized by the local Animal Care Committee “Organismo Preposto al Benessere degli Animali” and by Italian Ministry of Health “Ministero della Salute” (DL 26/2014).

## Author Contributions

AG, IZ, SP, GC, LF performed the experiments and analyzed the data; AMC carried out the histological analysis; SBr and FM supervised RT-PCR and gene sequencing assays; AF supervised *in vitro* assays; RC and AL supervised the binding assays; SBe, EB and MT designed the research and supervised the experiments; AG, IZ, and SBe wrote the manuscript; all the authors critically revised the manuscript, finally approved it and agreed to be accountable for all aspects of the work.

## Funding

This work was supported by FFABR_2017_MIUR grants to SB, EB, and MT.

## Conflict of Interest Statement

The authors declare that the research was conducted in the absence of any commercial or financial relationships that could be construed as a potential conflict of interest.

## Abbreviations

CD, Crohn’s disease; CsA, cyclosporine A; DAI, disease activity index; DSS, dextran sulphate sodium; EphB, erythropoietin-producing hepatocellular carcinoma type B; FCS, fetal calf serum; HBSS, Hank’s Balanced Salt Solution; HTAB, hexadecylthrimethyl-ammoniumbromide; IBDs, inflammatory bowel diseases; IL-1β, interleukin-1β; MLN, mesenteric lymph node; MPO, myeloperoxidase; MS, macroscopic score; PI, propidium iodide; PMA, phorbol 12-myristate 13-acetate; RCF, relative centrifugal force; RT-PCR, reverse transcription-polymerase chain reaction; SDS-PAGE, sodium dodecyl sulfate polyacrylamide gel electrophoresis; TCR, T-cell receptor; TNBS, 2,4,6-TriNitroBenzeneSulfonic acid; TNFα, tumor necrosis factor α; UC, ulcerative colitis.
