# Supplementary material for: Targeting the Eph/Ephrin System as Anti-Inflammatory Strategy in IBD
Source: Front Pharmacol. 2019 Jun 17;10:691. doi: 10.3389/fphar.2019.00691 (PMC6606944; doi:10.3389/fphar.2019.00691)
Supplement: Supplementary file 1 [file DataSheet_1.pdf]

Supplementary Table 1: Effects of IgG1-Fc on TNBS-induced inflammatory responses
Disease Activity Index (DAI1-4), macroscopic score, colonic length and thickness, colonic and lung MPO activity assessed in TNBS-treated mice administered with vehicle (Control) (n=10-12 independent values) or IgG1-Fc 10μg/kg s.c. (n= 6 independent values). *P<0.05 vs. C mice, one-way ANOVA followed by Bonferroni's post-test.

	Control	IgG1-Fc
DAI 1	0	0
DAI 2	2.7±0.1	2.7±0.3
DAI 3	3.8±0.1	3.5±0.4
DAI 4	4.8±0.2	3.7±0.6
Macroscopic score	3.5±0.3	2.3±0.4
Colonic length (cm)	5.2±0.1	6.2±0.2*
Colonic thickness (mg/cm)	61.7±2.3	60.7±2.0
Colonic MPO activity (U/g)	67.9±16.6	60.7±23.0
Lung MPO activity (U/g)	67.4±10.8	33.3±9.5

Supplementary Table 2: Effects of EphB4 and CsA on viability of splenic mononuclear cells

Percent viability of cultured mononuclear cells, assessed through trypan blue exclusion assay, after 24h incubation with vehicle, CsA 1μg/ml or EphB4 (10-100ng/ml) in the presence of PMA and ionomycin (n=3-4 independent values).

Treatment		Viability (%)
Vehicle		96±3
CsA (μg/ml)	1	90±8
EphB4 (ng/ml)	10	98±1
	100	98±1

Supplementary Figure 1: Effects of EphB4 on TNBS-induced inflammatory responses

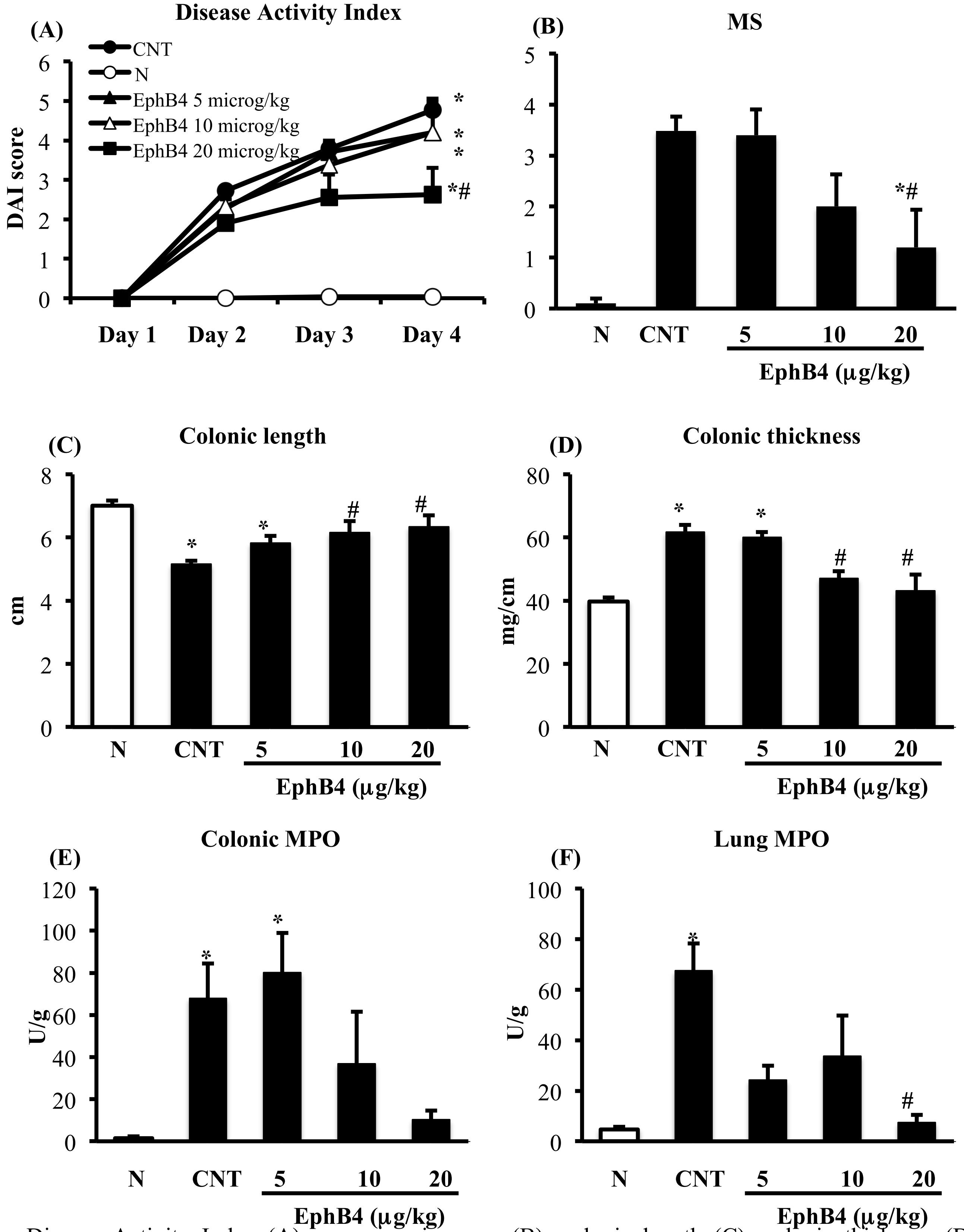

Disease Activity Index (A), macroscopic score (B), colonic length (C), colonic thickness (D), colonic MPO (E) and lung MPO (F) activity assessed in vehicle-treated normal mice (N) and in TNBS-treated mice administered with vehicle (CNT) or EphB4 5, 10, 20 μg/kg (n=5-12 independent values per group). *P<0.05 vs. N mice; #P<0.05 vs. CNT mice; one-way ANOVA followed by Bonferroni's post-test.

Supplementary figure 2: DSS did not induce changes in spleen and MLN T cells profile

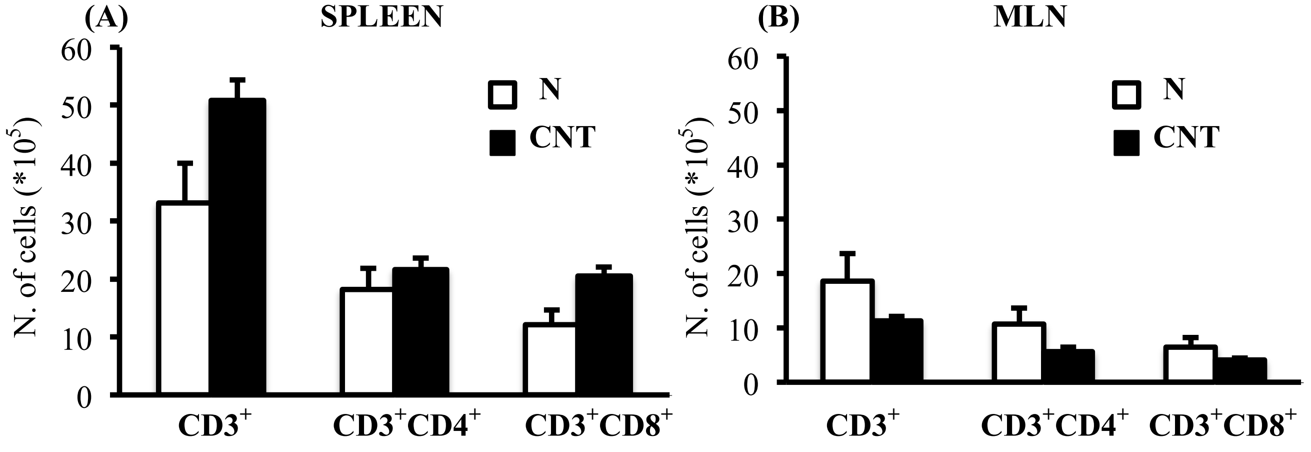

Number of T lymphocytes ($CD3^+$), $CD4^+$ and $CD8^+$ T lymphocytes in the spleen (A) and in MLN (B) excised from vehicle-treated normal mice (N – white bars) and DSS-treated mice administered with vehicle (CNT – black bars) (n=5-8 independent values per group).
